# Gene Profiling of *Mta1* Identifies Novel Gene Targets and Functions

**DOI:** 10.1371/journal.pone.0017135

**Published:** 2011-02-25

**Authors:** Krishna Sumanth Ghanta, Da-Qiang Li, Jeyanthy Eswaran, Rakesh Kumar

**Affiliations:** 1 McCormick Genomic and Proteomic Center, The George Washington University Medical Center, Washington, D.C., United States of America; 2 Department of Biochemistry and Molecular Biology, The George Washington University Medical Center, Washington, D.C., United States of America; The National Institute of Diabetes and Digestive and Kidney Diseases, United States of America

## Abstract

**Background:**

Metastasis-associated protein 1 (MTA1), a master dual co-regulatory protein is found to be an integral part of NuRD (Nucleosome Remodeling and Histone Deacetylation) complex, which has indispensable transcriptional regulatory functions via histone deacetylation and chromatin remodeling. Emerging literature establishes MTA1 to be a valid DNA-damage responsive protein with a significant role in maintaining the optimum DNA-repair activity in mammalian cells exposed to genotoxic stress. This DNA-damage responsive function of MTA1 was reported to be a P53-dependent and independent function. Here, we investigate the influence of *P53* on gene regulation function of *Mta1* to identify novel gene targets and functions of *Mta1*.

**Methods:**

Gene expression analysis was performed on five different mouse embryonic fibroblasts (MEFs) samples (i) the *Mta1* wild type, (ii) *Mta1* knock out (iii) *Mta1* knock out in which *Mta1* was reintroduced (iv) *P53* knock out (v) *P53* knock out in which *Mta1* was over expressed using Affymetrix Mouse Exon 1.0 ST arrays. Further Hierarchical Clustering, Gene Ontology analysis with GO terms satisfying corrected p-value<0.1, and the Ingenuity Pathway Analysis were performed. Finally, RT-qPCR was carried out on selective candidate genes.

**Significance/Conclusion:**

This study represents a complete genome wide screen for possible target genes of a coregulator, Mta1. The comparative gene profiling of *Mta1* wild type, *Mta1* knockout and *Mta1* re-expression in the *Mta1* knockout conditions define “bona fide” *Mta1* target genes. Further extensive analyses of the data highlights the influence of *P53* on *Mta1* gene regulation. In the presence of *P53* majority of the genes regulated by *Mta1* are related to inflammatory and anti-microbial responses whereas in the absence of *P53* the predominant target genes are involved in cancer signaling. Thus, the presented data emphasizes the known functions of *Mta1* and serves as a rich resource which could help us identify novel *Mta1* functions.

## Introduction

Gene expression is central to variety of fundamental cellular processes that governs the growth and proliferation in mammalian cell. It is a highly regulated process that is governed by transcription factors and their co-regulators in the target gene chromatin [1]–[3]. Co-regulators are emerging transcription factor (TF) family that stringently controls the actions of almost “all” nuclear receptors (NRs) through direct binding to NRs rather than binding to DNA [1]–[4]. Recent literature highlights the significance of coregulators in induction or repression of gene transcription [5], [6]. In fact, the coregulators either function as enzymes that are essential for gene expression or they regulate other coregulators through diverse mechanisms [6]–[10]. There has been a tremendous focus on understanding the molecular mechanism of coregulators due to their crucial regulatory role in almost “all” TF-dependent gene expression and nuclear receptors dependent functions in several tissues such as breast, ovary, prostate, gastrointestinal, pancreatic and lungs [5], [11], [12]. More interestingly, many cancers over express “growth coactivators” that allow the cancer cell to hijack these molecules which consequently results in rapid proliferation, malignant process and rapid metastasis.

One emerging group of chromatin modifiers and coregulators is the metastasis-associated protein (MTA) family. This family comprises three different known genes (*MTA1*, *MTA2*, and *MTA3*) and is an integral part of the NuRD (Nucleosome Remodeling and Histone Deacetylation) complex that has indispensable transcriptional regulatory functions via histone deacetylation and chromatin remodeling [13], [14]. MTA1, the founding member of the MTA family was initially identified through differential screening of a cDNA library from rat metastatic breast tumors as an upregulated gene [15], [16]. Subsequent studies found MTA1 to be widely up-regulated in various human cancers and established to be involved in tumorigenesis, tumor invasion, and metastasis [13], [17]. Owing to its name as a coregualtor the repressor function of MTA1 is observed through its direct interaction with ERα [18] and HDACs which represses estrogen-responsive element (ERE) transactivation activity in a HDAC-sensitive manner that promotes the development of hormone-independent growth of breast cancer cells. In addition, MTA1-NuRD complex was also reported to negatively regulate BRCA1 transcription by physically associating with an atypical estrogen-responsive element (ERE) on the BRCA1 promoter [19]. The transcription activator function of MTA1 is evident from reports showing the stimulation of breast cancer-amplified sequence 3 (BCAS3) [20] and paired box gene 5 (Pax 5) [21] promoters mainly through the interaction with RNA polymerase II. It is noteworthy that the MTA family members exist in distinct NuRD complexes, and functional redundancy is lacking among MTA family members [22].

Further, recent studies from this laboratory have discovered for the first time that MTA1 is a bona-fide DNA-damage responsive protein due to the induction of intracellular levels of MTA1 by ionizing radiation (IR) and an integral component of DNA damage response and contributes to double-strand DNA break repair [23]. One of the main DNA damage responsive mechanisms employed by MTA1 is through direct interaction and controlling the stability of P53 [24]. P53 is a well-studied tumor suppressor protein which plays a central role in preserving the genomic integrity in response to DNA damage, through inhibiting its ubiquitination by E3 ubiquitin ligases, mouse double minute 2 (Mdm2) and constitutive photomorphogenic protein 1 (COP1), thereby regulating the P53-dependent DNA repair [25]–[27]. Interestingly, these events could be reversed by MTA1 reintroduction, indicating that MTA1 interjects into the P53-dependent DNA repair [24]. This identified interplay between oncogene (MTA1) and tumor suppressor (P53) exemplifies the highly complex check point mediated “failsafe mechanism” that controls the mitogenic signaling. Moreover, studies from our laboratory illustrated the participation of MTA1 in a P53-independent DNA damage response. MTA1-histone deacetylase 2 (HDAC2) complexes recruit P53-independent transcriptional corepressor, p21WAF1 [28], [29] onto two selective regions of the p21WAF1 promoter which increases p21WAF1 binding to proliferating cell nuclear antigen (PCNA). This decreases the nuclear accumulation of PCNA in response to ionizing radiation [30]. MTA1 is also shown to be stabilized by UV radiation in ATR (Ataxia teleangiectasia and Rad3-related) kinase dependent manner and there is a subsequent increase in MTA1 binding to ATR. However, depletion of MTA1 compromises the ATR-mediated Chk1 activation following UV treatment. Consequently, expression of MTA1 in P53-null cells results in increased induction of histone, γH2AX [31] foci and DNA double strand break repair and decreased DNA damage sensitivity following ionizing radiation treatment. These early studies linking the MTA/NuRD complexes to DNA-damage response were further supported by more recent reports showing the recruitment of the NuRD complexes to the site of DNA damage [32], [33] Together, the P53-independent role of MTA1 in DNA damage response connects NuRD complex and DNA-damage response pathways [34].

To further characterize the potential mechanism for the functional role of MTA1 in DNA repair, we performed a complete gene profiling study to identify differences between the *MTA1* ^+/+^ and *MTA1* ^−/−^ mouse embryonic fibroblasts (MEFs) in the presence and absence of P53, using Affymetrix Mouse Exon 1.0 ST arrays. The comparative gene profiling of *Mta1* wild type, *Mta1* knock out and *Mta1* knockout in which *Mta1* was reintroduced conditions define “bona fide” Mta1 target genes such as *Egr2*, *Phf17*, and *Aw551984*. In depth analyses of the microarray data of all five samples that include the above three along with *P53* knockout and *P53* knockout with *Mta1* over expression clearly indicates influence of *P53* on *Mta1* gene regulation. Mta1 target genes are mostly involved in inflammatory and anti-microbial responses in the presence of P53 whereas the predominant target genes and functions identified appear to be related to cancer signaling in the absence of P53. Thus, we provide a complete gene profiling of *Mta1* which not only emphasizes the known functions of Mta1 but also acts as a guide to identify novel functions.

## Results

### Strategy to identify the possible target genes of MTA1 in a P53 dependent and independent manner

**Figure 1:**
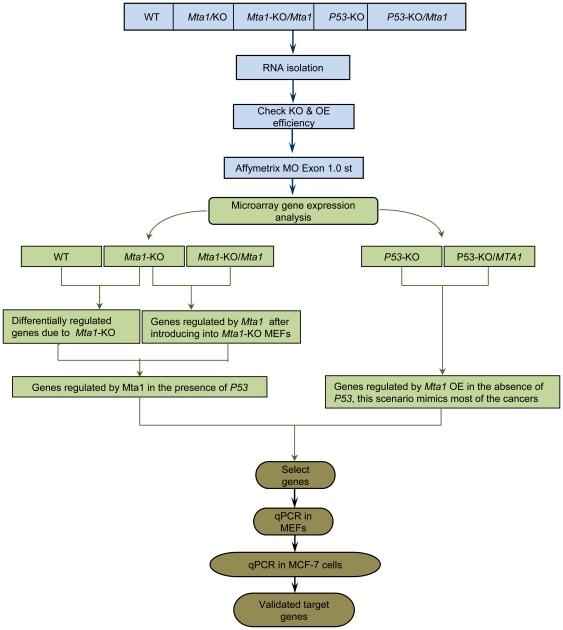
Schematic showing the experimental design of the study to identify the *Mta1* regulated genes with/without the effect of *P53*. RNA was extracted from all the samples Wild Type (WT), *Mta1* knock out (*Mta1-*KO), *Mta1* Re-expression in the *Mta1* knock out MEFS (*Mta1*-KO/*Mta1*), P53 knock out (*P53*-KO), *Mta1* over expression (OE) in the *P53* knock out MEFs ( *P53*-KO/*Mta1*). cDNA was prepared, processed and hybridized onto the Affymetrix Mouse Exon 1.0 ST arrays followed by the data analysis. Samples were compared to identify the differentially regulated genes and in turn the genes regulated by *Mta1* in *p53* dependent/independent manner and irrespective of *P53* status. Candidate genes were selected; these genes were validated using RT-qPCR assays in MEFs followed by RT-qPCR assays in MCF-7 human breast cancer cell line with the human homologs of the candidate mouse genes.

The aim of the study is to identify the genes that are regulated by *Mta1* in P53 dependent and independent manners. The detailed schematic of the strategy followed to identify the genes is shown in Figure 1. Murine Embryonic Fibroblasts (MEFs) from wild type, *Mta1* knockout [35] and *P53* knockout mice embryos [36] were isolated and cultured to obtain five different types of samples, each in triplicates, for the identification of the genes that are regulated by *Mta1* with/without the *P53* background. The sample sets are as follows: 1) *Mta1*-Wildtype (WT), 2) *Mta1*-knockout (*Mta1*-KO) [23], 3) *Mta1* transfected into the *Mta1*-knock out MEFs (*Mta1*-KO/*Mta1*), 4) *P53*-Knock (*P53*-KO) and 5) *Mta1* over expressed in the *P53*-Knockout MEFs (*P53*-KO/*Mta1*) [30]. The protein levels of Mta1 in all the five samples are compared using Western blot probed with Mta1 antibody (Figure S1). Total RNA was isolated from the MEFs, cDNA was prepared, processed and hybridized onto Affymetrix Mouse Exon 1.0 ST arrays. The gene expression data from all the samples were obtained, quality control steps were performed and the data was analyzed using GeneSpring GX 10.0.2 (Agilent Technologies).

Genes regulated by *Mta1* under different conditions were identified by performing gene expression analysis followed by the Gene Ontology analysis. Ingenuity Pathways Analysis (IPA) (Ingenuity Systems, Inc) was used to identify top 15 statistically significant functions of the differentially regulated genes and top 15 canonical pathways in which these genes could play a role. Candidate genes were selected based upon the functions and our laboratory interests. RT-qPCR assays were performed to validate the microarray gene regulation of the selected candidate genes. The differential regulation of the candidate genes was confirmed using MEFs and the MCF7 human breast cancer cell line for the respective human homologs. Using this strategy, we have identified a set of genes which, we believe, are regulated by *Mta1* in *P53* dependent and independent manners, thus, providing overall gene profiling of *Mta1*.

### Quality Control and Gene Expression Analysis

**Figure 2:**
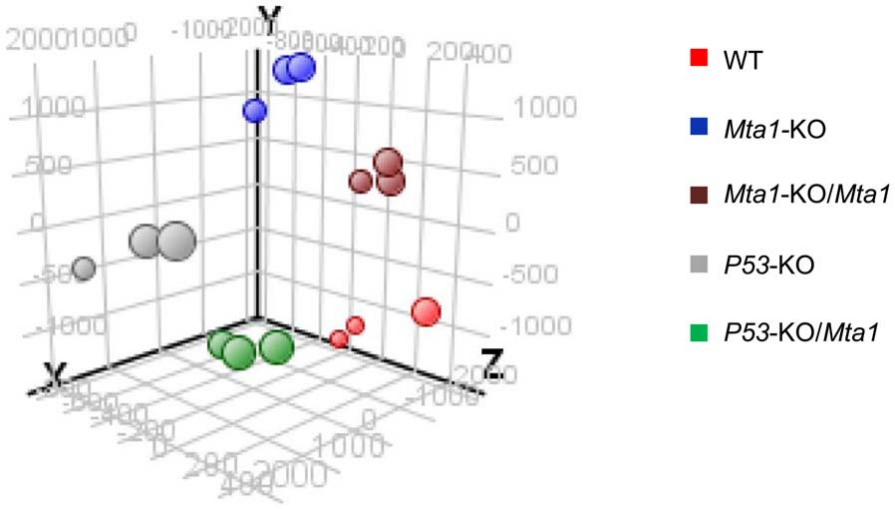
Principle Component Analysis as a quality control. All the sample sets (each in triplicates) were plotted in the 3-D PCA-plot. Out of the 4 total PCA components, components 1, 2 & 4 were plotted on columns X, Y & Z respectively.

We generated cDNAs from the total RNA of the corresponding MEFs and three biological replicates for each sample were hybridized. The raw data was obtained and normalized log2 ratio values were used to identify the differential gene expression levels across different samples. The quality control of all the samples and the replicates was analyzed by unsupervised Principle Component Analysis (PCA) method in which each of the samples, in a different color, was plotted in three dimensional (3D) space with components 1, 2 & 4 along X, Y & Z axes respectively. Figure 2 shows the PCA-plot with replicates of each sample clustered close to each other illustrating the similarity between the sample replicates. We performed unpaired t-test with a p-value less than 0.05 and Benjamini Hochberg false discovery rate (FDR) was applied for the multiple corrections to filter the false positives so that the statistically significant genes could be obtained.

### Genes regulated by *Mta1* in the presence of *P53* in MEFs

The initial comparison between the *Mta1* wild type, the *Mta1* knock out and *Mta1*-re-expression in knockout MEFs was performed. Although differential expression of the genes between *Mta1* wild type and *Mta1* knock out was reported by us earlier [30], our aim of this study is to identify the genes differentially regulated among *Mta1* wild type, *Mta1* knock out and *Mta1*-KO in which *Mta1* WT was reintroduced. Therefore, we have used the data from Li et al, (2010) for comparison. When *Mta1* WT and knockout conditions were compared, 1124 genes were reported with a fold change ≥±2.0 and with the p-value<0.05. These 1124 genes were either directly or indirectly regulated by *Mta1*. Top 25 differentially expressed Affymetrix probes are shown in Table 1 (complete list is shown in Table S1, statistically summary is shown in Table S2). It is noteworthy that a gene encoding epidermal growth factor-containing fibulin-like extra cellular matrix protein 1 (*EFEMP1*) is identified to be up-regulated by 100 fold with the highest fold change regulation in the *Mta1* knockout MEFs when compared with the wild type *Mta1* MEFs. Interestingly, Sadr-Nabavi et al., (2009) reported a reduction in RNA and protein levels of EFEMP1 in human sporadic breast cancer tissues [37] which imply that over expression of *Mta1* could lead to down regulation of *EFEMP*. In addition, we also found that the gene encoding CD24/HSA had the maximum fold change among the down regulated genes. CD24 is a cell adhesion molecule that is expressed on the surface of the infected cells [38].

**Table 1:** Top 25 differentially expressed Affymetrix Mouse Exon 1.0 St Array probe sets in MEFs between the wild type and *Mta1* knock out.

refseq	Gene Symbol	FC	Reg	Gene Description
NM_146015	*Efemp1*	100.24	up	epidermal growth factor-containing fibulin-like extracellular matrix protein 1
NM_009099	*Trim30*	79.45	up	tripartite motif-containing 30
NM_133871	*Ifi44*	79.08	up	interferon-induced protein 44
NM_009252	*Serpina3n*	72.25	up	serine (or cysteine) peptidase inhibitor, clade A, member 3N
NM_008331	*Ifit1*	71.62	up	interferon-induced protein with tetratricopeptide repeats 1
NM_015784	*Postn*	49.40	up	periostin, osteoblast specific factor
NM_009846	*Cd24a*	47.95	down	CD24a antigen | predicted gene, EG621324
NM_134086	*Slc38a1*	42.67	down	solute carrier family 38, member 1
NM_001033767	*EG240327*	41.75	up	predicted gene, EG240327
NM_001111059	*Cd34*	40.98	down	CD34 antigen
NM_011409	*Slfn3*	34.62	up	schlafen 3
NM_001085522	*RP24-320O9.1*	32.71	up	novel KRAB box containing protein
NM_010681	*Lama4*	30.71	up	laminin, alpha 4
NM_007833	*Dcn*	30.43	up	decorin
NM_009251	*Serpina3g*	30.43	up	serine (or cysteine) peptidase inhibitor, clade A, member 3G
NM_023386	*Rtp4*	28.17	up	receptor transporter protein 4
NM_010917	*Nid1*	27.87	up	nidogen 1 | similar to Nid1 protein
NM_008409	*Itm2a*	26.76	up	integral membrane protein 2A
NM_011854	*Oasl2*	26.58	up	2′-5′ oligoadenylate synthetase-like 2
NM_008489	*Lbp*	26.45	up	lipopolysaccharide binding protein
NM_019955	*Ripk3*	25.19	up	receptor-interacting serine-threonine kinase 3
NM_025711	*Aspn*	23.86	up	asporin
NM_025949	*Rps6ka6*	23.80	down	ribosomal protein S6 kinase polypeptide 6
NM_025961	*Gatm*	23.37	down	glycine amidinotransferase (L-arginine:glycine amidinotransferase)
NM_001001892	*EG630499*	23.27	up	histocompatibility 2, K1, K region

Top 25 probe sets with fold change of 2.0 or more and FDR less than 0.05 are shown. Wild type cells are the control against the knock out cells as treatment.

**Figure 3:**
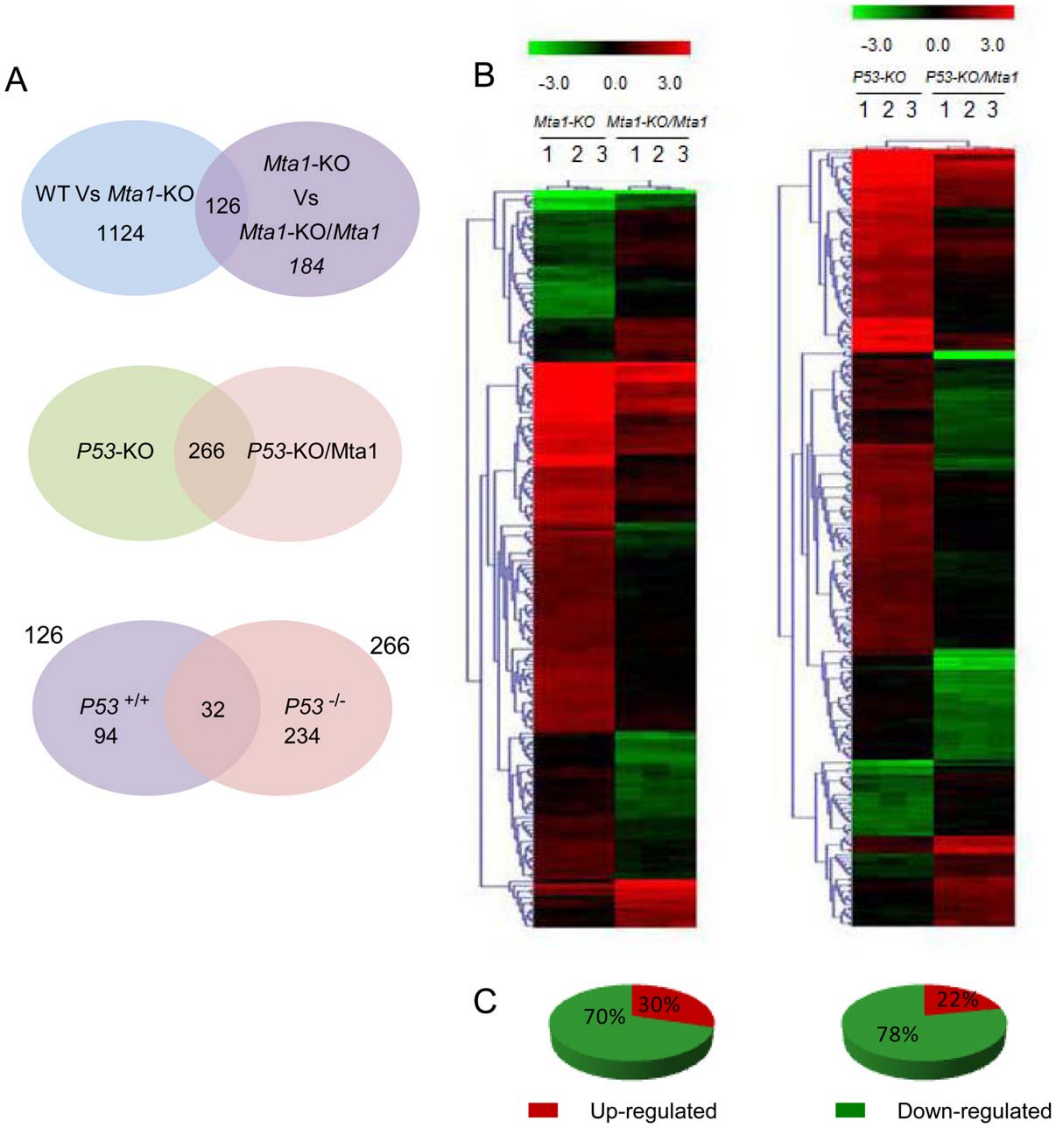
Genes regulated by *Mta1 *in the presence and absence of *P53*. **A**: Venn diagrams showing the number of genes that are identified to be genuinely regulated by *Mta1* in the presence and absence of *P53*. (i) The genes shown in WT vs. *Mta1*-KO (1124) are the genes affected by *Mta1* knock out and the genes that are present in *Mta1-*KO vs. *Mta1-*KO*/Mta1* (184) are affected due to re-expression of *Mta1* in the knockout MEFs. The genes that are differentially regulated in both the sets with opposite trends in the regulation are considered as the ‘bona fide’ genes regulated by *Mta1*. ii) Similarly, the intersection of *P53*-KO and *P53*-KO/*Mta1* represents the genes differentially regulated by *Mta1* over expression in the *P53-*KO MEFs. i.e. the genes regulated by *Mta1* in the absence of *P53*.This scenario of *Mta1* over expression and *P53* knock out/mutation mimics most of the cancers iii) The Venn diagram between genes regulated by *Mta1* in the presence of *P53*, *126* and genes regulated by *Mta1* in the absence of *P53*, *266* gives the genes that are regulated by *Mta1* only in the presence of *P53*, irrespective of *P53* status and only in the absence of *P53* respectively. **B**: Heat maps representing hierarchical clustering of the differentially regulation, plotted using the log2 values of the genes with p-value≤0.05 (unpaired t-test) and with fold change of at least 2 between the comparisons *Mta1* knock out vs. *Mta1* knock out/*Mta1* and *P53* knock out vs. *P53* knock out/Mta1. Each column represents a sample plotted in triplicates and each row in the heat map represents a gene that is differentially regulated in that particular comparison of samples. The color scale represents the degree of expression of the gene, green being the lowly expressed (below −3.0) and red being (above +3.0) the highly expressed genes in the sample sets with black as the center of the scale at ‘0’. **C**: Pie diagrams showing the percentage of up-regulated and the down-regulated genes in each sample comparison. The red segment of the pie represents the up-regulated genes whereas; the green segments represent the down regulated genes.

When *Mta1* was transfected into the *Mta1* knock-out MEFs and compared against the *Mta1*-KO, 184 differentially regulated genes were identified. The top 25 differentially regulated genes based upon fold change are shown in Table 2 (the entire list is shown in Table S3 and statistical summary in Table S4). Out of these 184 genes, 126 genes were found to be present in WT Vs *Mta1-*KO as well (1124 genes, Figure 3A top panel). Majority of these 126 genes appear to restore their functioning when *Mta1* is transfected back into the *Mta1* knock out cells. Among these 126 genes except 6 genes, rest of them regained their expression levels similar to WT. Therefore these represent “bona fide” *Mta1* target genes (Table 3 and Table S5). Since *P53* is present in all the three samples they reflect the number of genes influenced by *MTA1* in the presence of *P53*.

**Table 2:** Top 25 differentially expressed Affymetrix Mouse Exon 1.0 St Array probe sets in MEFs between the *Mta1* knock out and *Mta1* knock out/*Mta1*.

refseq	Gene Symbol	FC	Reg	Gene Description
NM_134066	*Akr1c18*	14.98	up	aldo-keto reductase family 1, member C18
NM_025711	*Aspn*	9.58	down	asporin
NM_029803	*Ifi27*	8.86	down	interferon, alpha-inducible protein 27
NM_178737	*AW551984*	6.75	down	Mouse homolog of human gene *VWA5A*
NM_007621	*Cbr2*	6.01	down	carbonyl reductase 2
NM_011595	*Timp3*	5.67	up	tissue inhibitor of metalloproteinase 3
NM_027495	*Tmem144*	5.13	up	transmembrane protein 144
NM_029000	*Gvin1*	4.89	down	GTPase, very large interferon inducible 1
NM_013585	*Psmb9*	4.53	down	proteasome (prosome, macropain) subunit, beta type 9 (large multifunctional peptidase 2)
NM_028608	*Glipr1*	4.36	down	GLI pathogenesis-related 1 (glioma)
NM_023422	*Hist1h2bc*	3.91	down	histone cluster 1
NM_001097644	*Ccnyl1*	3.91	down	cyclin Y-like 1
NM_008630	*Mt2*	3.78	up	metallothionein 2
NM_008604	*Mme*	3.78	down	membrane metallo endopeptidase
NM_008728	*Npr3*	3.74	up	natriuretic peptide receptor 3
NM_009251	*Serpina3g*	3.73	down	serine (or cysteine) peptidase inhibitor, clade A, member 3G
NM_011979	*Vnn3*	3.68	down	vanin 3
NM_007817	*Cyp2f2*	3.65	down	cytochrome P450, family 2, subfamily f, polypeptide 2
NM_008185	*Gstt1*	3.64	down	glutathione S-transferase, theta 1
NM_054077	*Prelp*	3.58	down	proline arginine-rich end leucine-rich repeat
NM_053078	*D0H4S114*	3.57	down	DNA segment, human D4S114
NM_011579	*Tgtp*	3.50	down	T-cell specific GTPase
NM_012043	*Islr*	3.45	down	immunoglobulin superfamily containing leucine-rich repeat
NM_009846	*Cd24a*	3.32	up	CD24a antigen
NM_133903	*Spon2*	3.26	down	spondin 2, extracellular matrix protein

Top 25 probe sets with fold change of 2.0 or more and FDR less than 0.05 are shown. *Mta1* knock out cells are the controls and Mta1-KO/Mta1 is the treatment.

**Table 3:** Bona fide *Mta1* regulated genes in the presence of *P53* identified from the Affymetrix Mouse Exon 1.0 ST Arrays.

Gene Symbol	WT vs. *Mta1*-KO		*Mta1*-KO vs. *Mta1*-KO/*Mta1*	
	Fold Change	Regulation	Fold Change	Regulation
Cd24a	47.95	down	3.32	up
Iigp1*	41.75	up	3.23	down
Serpina3g*	30.43	up	3.73	down
Lbp*	26.45	up	2.51	down
Aspn*	23.86	up	9.58	down
Ifit3	22.29	up	2.25	down
Apod	20.15	up	2.89	down
Irgb10	18.67	up	2.2	down
Ccnyl1	18.12	up	3.91	down
Apol9a	17.31	up	2.37	down
Apol9b*	17.31	up	2.37	down
Oas1a	15.93	up	2.19	down
Zbp1	13.03	up	3.15	down
Smpdl3b	12.92	down	2.26	up
Islr*	12.85	up	3.45	down
Steap4*	12.37	up	2.55	down
Pcdhb9	12.31	up	2.22	down
Timp3	12.03	down	5.67	up
Ifi27	11.75	up	8.86	down
Irf7	11.17	up	2.04	down
Prelp*	11.12	up	3.58	down
Psmb9	11.05	up	4.53	down
AW551984	11.04	up	6.75	down
Pcdhb8	9.98	up	2.82	down
Tspan13	9.95	up	2.03	down
Il1rn	9.87	up	2.34	down
Gvin1	9.87	up	4.89	down
Lpl	9.54	up	2.36	down
Il1rl1*	9.08	up	2.32	up
Oas2	8.78	up	2.98	down
Trim12	8.32	up	2.04	down
Adam23	8.32	up	2.74	down
Ednra	8.06	up	2	down
A4galt*	7.21	up	2.85	down
Tap1	7	up	2.89	down
Ened	6.88	up	2.31	up
Vnn3	6.81	up	3.68	down
Dhx58	6.49	up	2.26	down
Mgp	6.29	up	2.64	down
Ces1	6.14	up	2.9	down
Slc16a4	5.97	up	3	down

*Genes (32) regulated by *Mta1* irrespective of *P53* status. Figure 3A (lower panel).

Note: Continued in Table S5.

### Genes regulated by *Mta1* in a *P53-*independent manner

Here we identified the genes that are regulated by *Mta1* in a *P53-*independent manner. Differential gene expression analysis was performed by comparing the *P53* knock out cells and *P53* knock out/*Mta1* over expressed cells [30]. All the genes with *p*-value<0.05 and fold change expression ≥±2.0 were considered as statistically significant. The statistical analysis of the data generated a set of 266 genes, top 25 differentially regulated genes are shown in Table 4 (entire list is shown in Table S6 and statistical summary in Table S7) and the Venn diagram in Figure 3A middle panel shows the number of genes differentially regulated. When Bona fide *Mta1* regulated genes (126) were compared with *p53* independent regulated genes of *Mta1*, 32 genes were shown to be regulated by *Mta1* irrespective of *p53* status (Figure 3A lower panel, these genes are marked with asterisks in Table 3 and Table S5). Since, both the samples are *P53-*knockout MEFs, with one of them carrying over expression of *Mta1*; the differentially expressed genes were regulated by *Mta1* independent of *P53*.

**Table 4:** Top 25 differentially expressed Affymetrix Mouse Exon 1.0 St Array probe sets between the *P53* knock out MEFs and the *P53* knock out MEFs in which *Mta1* is over expressed.

refseq	Gene Symbol	FC	Reg	Gene Description
NM_008489	*Lbp*	26.89	down	lipopolysaccharide binding protein
NM_015784	*Postn*	14.70	down	periostin, osteoblast specific factor
NM_008607	*Mmp13*	13.37	down	matrix metallopeptidase 13
NM_008760	*Ogn*	12.46	down	osteoglycin
NM_011339	*Cxcl15*	12.39	down	chemokine (C-X-C motif) ligand 15
NM_008491	*Lcn2*	9.68	down	lipocalin 2
NM_001014423	*Abi3bp*	9.31	down	ABI gene family, member 3 (NESH) binding protein
NM_199468	*Zcchc5*	7.85	down	zinc finger, CCHC domain containing 5
NM_011704	*Vnn1*	7.22	down	vanin 1
NM_009373	*Tgm2*	7.22	down	transglutaminase 2, C polypeptide
NM_054098	*Steap4*	7.06	down	STEAP family member 4
NM_011315	*Saa3*	6.70	down	serum amyloid A 3
NM_010582	*Itih2*	6.51	down	inter-alpha trypsin inhibitor, heavy chain 2
NM_009144	*Sfrp2*	6.46	down	secreted frizzled-related protein 2
NM_010809	*Mmp3*	6.39	down	matrix metallopeptidase 3
NM_008728	*Npr3*	5.80	down	natriuretic peptide receptor 3
NM_172463	*Sned1*	5.70	down	sushi, nidogen and EGF-like domains 1
NM_022814	*Svep1*	5.62	down	sushi, von Willebrand factor type A, EGF and pentraxin domain containing 1
NM_007621	*Cbr2*	5.55	down	carbonyl reductase 2
NM_009251	*Serpina3g*	5.53	down	serine (or cysteine) peptidase inhibitor, clade A, member 3G
NM_025711	*Aspn*	5.47	down	asporin
NM_144938	*C1s*	5.23	down	complement component 1, s subcomponent
NM_031167	*Il1rn*	5.15	down	interleukin 1 receptor antagonist
NR_001592	*H19*	5.14	down	
NM_030601	*Clca2|Clca1*	4.83	down	chloride channel calcium activated 2 | chloride channel calcium activated 1

Top 25 probe sets with fold change of 2.0 or more and FDR less than 0.05 are shown. *P53-*KO is considered as the control and *P53*-KO/*Mta1* is considered as the treatment.

### Hierarchical clustering analysis reveals influence of P53 on MTA1 gene regulation

Further, we performed the hierarchical clustering analysis with the genes that were differentially regulated between the *Mta1*-KO vs. *Mta1*-KO/*Mta1*, and *P53*-KO vs. *P53*-KO/*Mta1*. The normalized log2 ratio values of the differentially regulated genes in each comparison were used to obtain the heat maps (Figure 3B). The gene leaf nodes were optimized in the heat maps representing the differential regulation of the genes between the samples. Color scale of the heat map depicts red as the highly expressed, green as low expressed and black as intermittent level of gene expression. Figure 3B shows the heat maps of all the comparisons, *Mta1*-KO with *Mta1*-KO/*Mta1* and *P53*-KO with *P53*-KO/*Mta1* representing the differential expression of the genes. We found that 64% of the differentially regulated genes were up-regulated in WT vs. *Mta1*-KO where as it greatly reduced to 30% in *Mta1*-KO vs. *Mta1*-KO/*Mta1* followed by further reduction to 22% in *P53*-KO vs. *P53*-KO/*Mta1*. In the case of differentially regulated genes that were down regulated, the percentage of down regulated genes was 36% in WT vs. *Mta1*-KO, 70% in *Mta1*-KO vs. *Mta1*-KO/*Mta1*and 78% in *P53*-KO vs. *P53*-KO/*Mta1*. In summary, we observe 78% of genes down regulated by *Mta1* due to the absence of P53 whereas about 64% of *Mta1* regulated genes were up regulated in the presence of *P53* (Figure 3C).

### Gene Ontology analysis shows the genes involved in cellular functions regulated by *Mta1* in the presence/absence of *P53*

**Figure 4:**
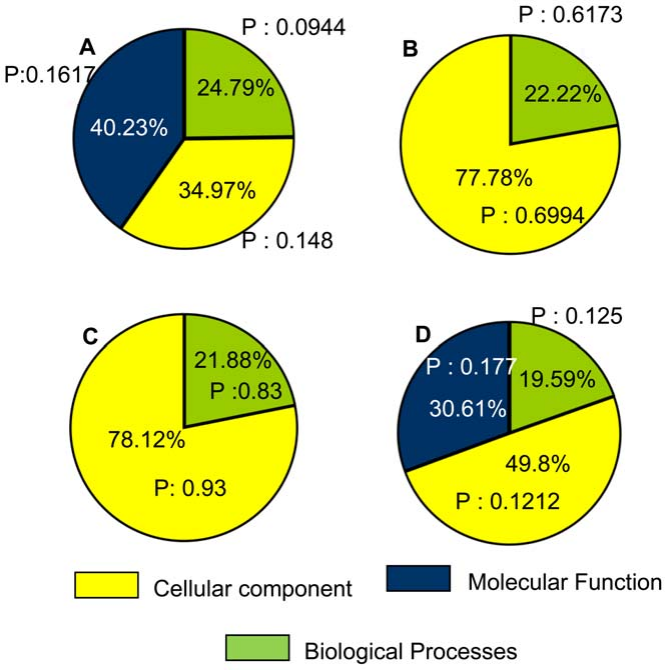
Gene Ontology (GO) analysis of was performed using Gene Spring GX 10.0.2 (Agilent technologies Inc and Strand Life Sciences Pvt Ltd). With the gene set comparisons that were described in the figure 3A GO analysis was performed. Pie chart A shows the statistically significant (P<0.1) differentially regulated genes between wild type and *Mta1-*KO samples matching with three broad GO terms (Cellular components, Biological process and Molecular Function), Pie chart B (*Mta1-*KO and *Mta1*-KO/*Mta1*) shows the GO terms matching with the differentially regulated genes in this comparison. Pie chart C and pie chart D show the same for bona fide *Mta1* regulated genes in the presence of *P53* (126) and *P53*-KO vs. *P53*-KO/*Mta1* (genes regulated by *Mta1* in the absence of *P53*,*266*) respectively.

To further investigate the functions of the genes regulated by *Mta1*, we performed Gene Ontology (GO) analysis on all the sets of genes that are regulated by *Mta1* with/without *P53* background with a *p*-value cutoff set to 0.1. The possible functions of the gene sets were broadly classified, in GO program, into three categories namely i) Cellular Component ii) Molecular Function and iii) Biological Process. We found that the 40.23% of the differentially regulated genes in the comparison of WT vs. *Mta1*-KO, with *p*-value: 0.1617 related to molecular function, followed by 34.97% with *p*-value 0.148 connected to Cellular Component and 24.79% of the genes with *p*-value 0.0944 were associated with the biological process (Figure 4A). In contrast to the above, when *Mta1*-KO and *Mta1*-KO with reintroduced *Mta1* were compared there were no genes that match the GO terms in the Molecular functioning, whereas majority of them (77.78%, *p-*value: 0.6994) were related to the cellular component (Figure 4B). With the corrected *p*-value cut off 0.1 and in the presence of P53, *Mta1* doesn't regulate any genes responsible for molecular functioning. The majority of the genes (78.12%) are associated with Cellular Component and 21.88% are associated to Biological Process (Figure 4C). In contrast to the above, in. the absence of *P53*, 49.8% (*p*-value: 0.1212) differentially expressed genes were linked to the Cellular Component, 30.61% (*p*-value: 0.1777) to Molecular Function and 19.59% (*p*-value: 0.1253) related to Biological Process (Figure 4D). Tables with GO terms for all the above mentioned comparisons are shown in Table 5, 6, 7 and 8 respectively. Table 5 and Table 8 are continued as Tables S8 and S9 respectively.

**Table 5:** GO Analysis of the genes differentially regulated between the WT & *Mta1*-KO with ≥2.0 fold change.

GO ACCESSION	GO Term	p-value	Corrected p-value	Count in Selection	% Count in Selection	Count in Total	% Count in Total
GO:0005576	extracellular region	1.87E-27	4.82E-22	286	44.83	2627	14.72
GO:0044421	extracellular region part	2.27E-25	2.92E-20	244	38.24	2157	12.08
GO:0005615	extracellular space	2.75E-23	2.36E-18	229	35.89	2038	11.42
GO:0007155	cell adhesion	2.10E-17	1.08E-12	78	12.23	541	3.03
GO:0022610	biological adhesion	2.10E-17	1.08E-12	78	12.23	541	3.03
GO:0006955	immune response	1.17E-14	5.02E-10	59	9.25	405	2.27
GO:0031012	extracellular matrix	9.71E-14	3.57E-09	50	7.84	279	1.56
GO:0005578	proteinaceous extracellular matrix	2.06E-13	6.64E-09	49	7.68	275	1.54
GO:0005488	binding	2.37E-13	6.80E-09	446	69.91	10687	59.87
GO:0005515	protein binding	7.79E-12	2.01E-07	325	50.94	5094	28.54
GO:0002376	immune system process	8.35E-11	1.96E-06	59	9.25	658	3.69
GO:0009653	anatomical structure morphogenesis	4.52E-09	9.71E-05	28	4.39	1002	5.61
GO:0005509	calcium ion binding	5.37E-09	9.89E-05	88	13.79	791	4.43
GO:0032502	developmental process	5.06E-09	9.89E-05	84	13.17	2682	15.03
GO:0048513	organ development	7.10E-09	1.14E-04	28	4.39	1339	7.50

Note: Continued in Table S8.

**Table 6:** GO Analysis of the genes differentially regulated between the *Mta1*-KO and *Mta1*- KO/Mta1 probes with ≥2.0 fold change.

GO ACCESSION	GO Term	p-value	corrected p-value	Count in Selection	% Count in Selection	Count in Total	% Count in Total
GO:0044421	extracellular region part	2.56E-12	0.00	53	72.60	2157.00	12.08
GO:0005615	extracellular space	1.18E-11	0.00	53	72.60	2038.00	11.42
GO:0005576	extracellular region	2.18E-11	0.00	61	83.56	2627.00	14.72
GO:0006955	immune response	1.01E-07	0.01	16	21.92	405.00	2.27
GO:0022610	biological adhesion	6.43E-06	0.28	15	20.55	541.00	3.03
GO:0007155	cell adhesion	6.43E-06	0.28	15	20.55	541.00	3.03

**Table 7:** GO Analysis of the bona fide *Mta1* regulated genes in the presence of *P53* with ≥2.0 fold change.

GO ACCESSION	GO Term	p-value	corrected p-value	Count in Selection	% Count in Selection	Count in Total	% Count in Total
GO:0044421	extracellular region part	2.30E-12	2.97E-07	43	78.18	2157	12.08
GO:0005615	extracellular space	1.48E-12	2.97E-07	43	78.18	2038	11.42
GO:0005576	extracellular region	8.02E-12	6.89E-07	48	87.27	2627	14.72
GO:0006955	immune response	8.12E-08	0.0052	13	23.64	405	2.27

**Table 8:** GO Analysis of the bona fide *Mta1* regulated genes in the absence of *P53* with ≥2.0 fold change.

GO ACCESSION	GO Term	p-value	corrected p-value	Count in Selection	%Count in Selection	Count in Total	% Count in Total
GO:0005576	extracellular region	8.45E-38	2.18E-32	122	89.05	2627	14.72
GO:0044421	extracellular region part	7.08E-29	9.12E-24	99	72.26	2157	12.08
GO:0005615	extracellular space	2.37E-23	2.03E-18	88	64.23	2038	11.42
GO:0005578	proteinaceous extracellular matrix	1.84E-15	1.19E-10	27	19.71	275	1.54
GO:0031012	extracellular matrix	2.64E-15	1.36E-10	27	19.71	279	1.56
GO:0006954	inflammatory response	3.07E-08	0.00131978	7	5.11	204	1.14
GO:0007155	cell adhesion	1.83E-07	0.00522946	21	15.33	541	3.03
GO:0022610	biological adhesion	1.83E-07	0.00522946	21	15.33	541	3.03
GO:0005509	calcium ion binding	7.26E-07	0.0170145	30	21.90	791	4.43
GO:0009605	response to external stimulus	6.98E-07	0.0170145	7	5.11	465	2.61
GO:0044268	multicellular organismal protein metabolic process	1.99E-06	0.0264874	5	3.65	16	0.09
GO:0004714	transmembrane receptor protein tyrosine kinase activity	2.05E-06	0.0264874	7	5.11	61	0.34
GO:0030574	collagen catabolic process	1.99E-06	0.0264874	5	3.65	16	0.09
GO:0008237	metallopeptidase activity	1.68E-06	0.0264874	12	8.76	181	1.01
GO:0044259	multicellular organismal macromolecule metabolic process	1.99E-06	0.0264874	5	3.65	16	0.09

Note: Continued in Table S9.

### Ingenuity Pathways Analysis highlights the critical role of Mta1 in cancer signaling in the presence/absence of *P53*

**Figure 5:**
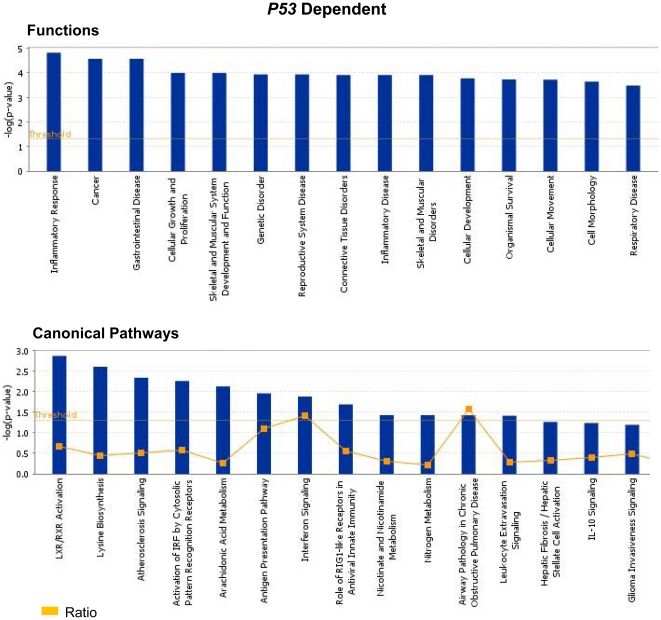
Ingenuity Pathway Analysis (Ingenuity Systems, Inc) of the genes that were regulated by *Mta1* in the presence of *P53* was performed. The significance of each function or canonical pathway is determined based upon the *p-*values determined using Right tailed Fisher's exact test and with threshold less than 0.05. The top 15 possible functions and canonical pathways of the genes regulated by *Mta1* in *P53* dependent manner are shown. Ratio of number of genes in a given pathway satisfying the cutoff and total number of genes present in that pathway was determined by IPA.

**Figure 6:**
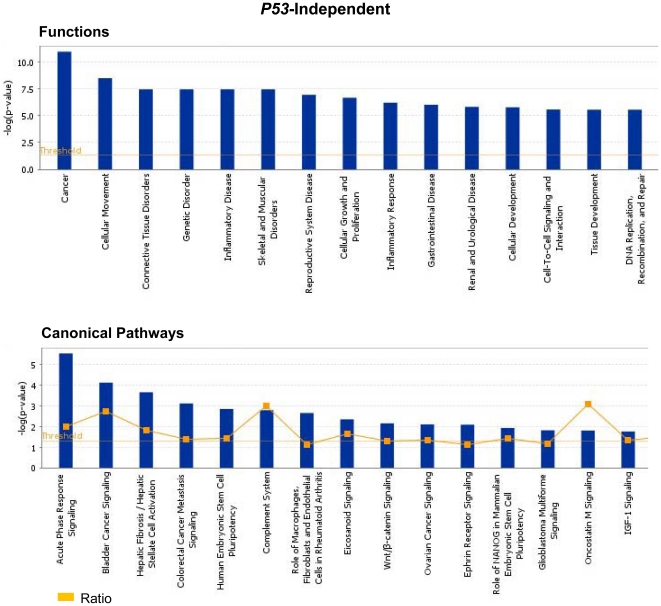
Ingenuity Pathway Analysis (Ingenuity Systems, Inc) of the genes that were regulated by *Mta1* in the absence of *P53* was performed. Fisher's exact test was used and threshold of 0.05 was set as the cutoff. Top 15 functions associated with the genes and the top 15 canonical pathways in which these genes might have a role are shown.

Ingenuity pathways analysis was performed on all the genes that were identified to be regulated by *Mta1* with/without *P53*. With *p*-value<0.05, Fischer's exact test was applied and we found top 15 significant functions and canonical pathways in which the genes regulated by *Mta1* might play a significant role. The most likely functions of the genes regulated by *Mta1* in the presence of *P53* are Inflammatory Response followed by Cancer and Gastrointestinal Diseases (Figure 5 upper panel). Top 15 canonical pathways of these genes were identified with the *p*-value<0.05 and threshold value of log (*p*-value): 0.05. The significant pathways which include LXR/RXR activation, Interferon signaling, Antigen presentation pathway and Activation of IRF by cytosolic pattern Recognition Receptors were shown in Figure 5 (lower panel). From these observations we propose that *Mta1* might have critical functional role in orphan nuclear receptor activation, inflammation and infections. Similarly, top 15 plausible functions of the genes regulated by *Mta1* in the absence of *P53* were identified and shown in Figure 6 (upper panel). The most significant function of the genes was found to be related to ‘Cancer’ followed by ‘Cellular Movement’ and ‘Connective Tissue Disorders’. The top 15 canonical pathways in which the genes might be involved were identified and shown in the Figure 6 (lower panel). The most significant canonical pathway identified is ‘Acute Phase response Signaling’ followed by Colorectal Cancer Metastasis Signaling, Hepatic fibrosis and ‘Bladder Cancer Signaling’. Clearly the genes regulated by *Mta1* in the absence of *P53* highlight the typical oncogenic character of *Mta1* and its possible major role in several cancers and oncogenic signaling pathways.

### The identified *Mta1* regulated candidate genes and their human homologs follow similar expression profile in MEFs and human breast cancer cell lines

**Figure 7:**
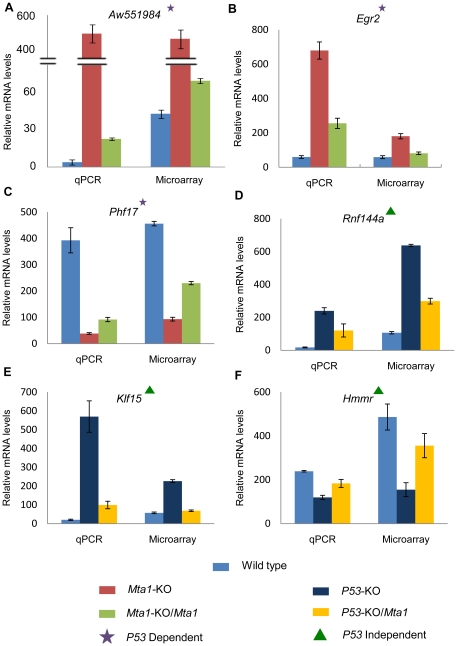
RT-qPCR validation of the microarray data showing the differential regulation of the selected genes in the MEFs. The RNA extracted from the fibroblasts of the wild type (light blue bars) mice was used as the control and the treatments were *Mta1*-KO (red bars) & *Mta1*-KO/*Mta1(green bars)*. In the presence of *P53* relative mRNA levels of the genes *Aw551984*, *Egr2*, *Phf17* were compared in all the three samples. The relative mRNA levels from microarray for the same sample sets were plotted and compared with the RT-qPCR. As expected, opposite trends of expression were observed between the knock out and re-expression models. To validate the genes regulated by *Mta1* in the absence of *P53* relative mRNA levels were compared among the three samples wild type (light blue bars), *P53*-KO (dark blue bars) & *P53*-KO/*Mta1*(yellow bars) for the genes *Hmmr*, *Klf15*, *Rnf144a*.The relative mRNA levels from the microarray were plotted and compared with RT-qPCR. Opposite trends of expression were observed between the *P53*-KO and *P53*-KO/*Mta1* treatments when compared with the wild type sample.

Based upon our analysis and our laboratory interest, some of the genes that were regulated by *Mta1* with/without *P53* back ground were selected for the validation using the RT-qPCR assays. Validations were first performed in the MEFs followed by the MCF7 human breast cancer cell line. The results showing relative mRNA levels, for the selected genes, are presented in Figure 7. The controls are compared with the treatments and if the trend of expression (up or down regulation) in qPCR is in agreement with the microarray gene expression, then the respective homologous genes were selected for further validation in the human breast cancer cell line (MCF7). *Aw551984* gene is the homolog of the human gene *VWA5A*, also known as BCSC-1. Monaco et al (1997) characterized and proposed that this gene could be a tumor suppressor [39], [40]. We found that both *Aw551984*/*VWA5A* were negatively regulated by *Mta1 in* MEFs and human breast cancer cells. In the case of *VWA5A*, the expression trend is similar in MEFs and MCF7 cells but the difference in expression levels between the WT and *MTA1* knockdown in the MCF7 cell line is marginal. Up regulation of this gene was observed in *Mta1*-KO and *MTA1-siRNA* knock down samples when compared to the wild type MEFs and MCF7cells respectively. Another candidate, early growth response protein 2 [encoded by *Egr2*] and its human homolog was found to follow similar trend in MEFs and MCF7 cell line with significant difference in expression levels when compared between wild type and *Mta1*-KO. Finally, *Phf17*, a known apoptosis promoter which may act as a renal tumor suppressor [41] is found to be down regulated in the *Mta1* knock out MEFs. As expected the expression pattern of all these candidates is in agreement with the above described microarray data. Together, the expression of these representative genes highlights the possible interplay between the tumor suppressors (*Aw551984*, *Egr2*), apoptotic protein *(Phf17)* and oncogene *(Mta1)*. The bar charts (7A, 7B, 7C) in Figure 7 show the expression levels of these three genes from the RT-qPCR assay and the Affymetrix microarray data.

We also selected candidate genes that are regulated by *Mta1*, in the absence of *P53*, from the microarray data and subjected them to the RT-qPCR validation. The relative expression levels of these genes in the controls and treatments are shown in the Figure 7 (7D, 7E, 7F). The data clearly shows the agreement between the microarray and the RT-qPCR results. We have chosen *Rnf144a* which contains RING figure motif and established to have role in protein-DNA and protein-protein interactions. We found that *Rnf144a* was upregulated in the absence of *P53* and over expression of *Mta1*. Another selected candidate was *Hmmr*, which encodes Hyaluronan mediated motility receptor protein that is found to be expressed in the breast tissue. It is notable that the *Hmmr* gene was found to be down regulated in *P53*-KO, whereas it was up regulated in *P53*-KO/*MTA1* when compared with the WT. The third candidate gene, *Klf15* which encodes a protein called kruppel like factor-15 was found to be highly up-regulated in *P53*-KO MEFs where as it is only weakly up-regulated in *P53*-KO/*Mta1*, when compared with the wild type. The expression levels from the RT-qPCR assay and the Affymetrix microarray data are in perfect agreement for all the chosen candidates in MEFs (Figure 7).

**Figure 8:**
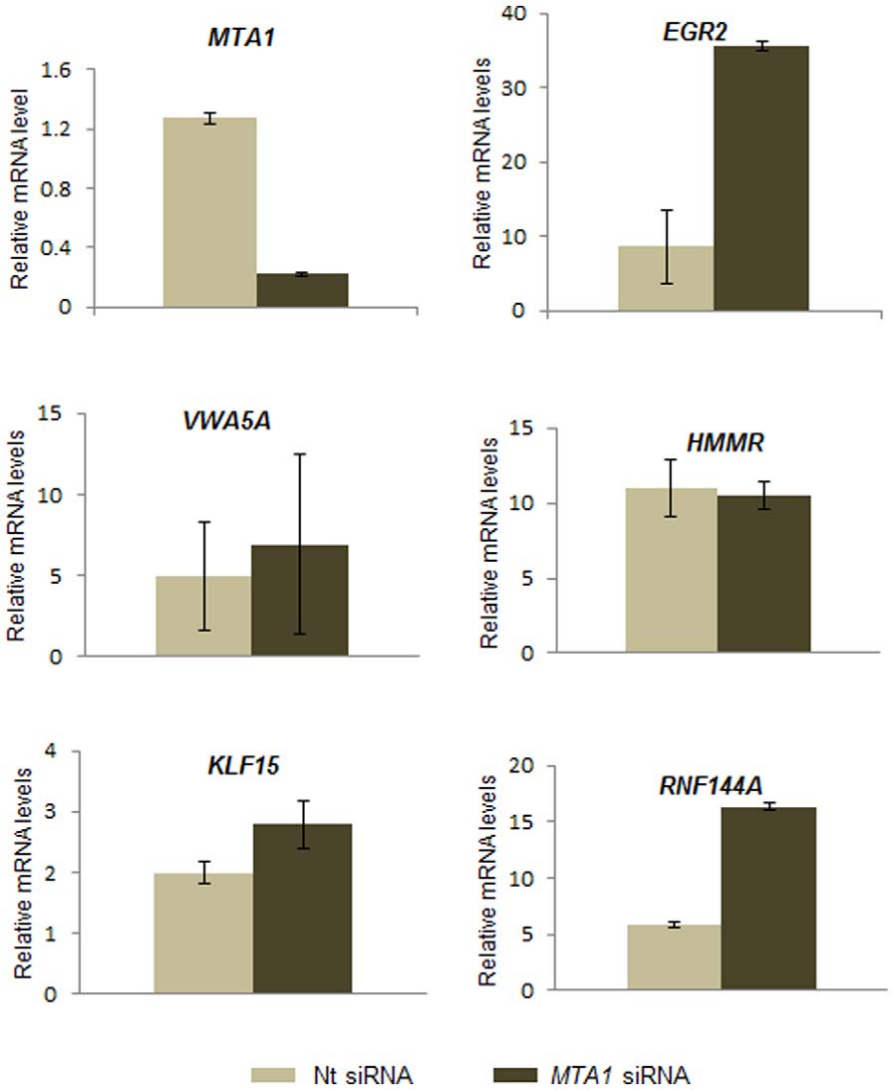
RT-qPCR was performed using the RNA obtained from the wild type MCF-7 cells and MCF-7 cells transfected with the *MTA1*-siRNA. The differential regulation of the selected genes (*EGR2*, *HMMR*, *KLF15*, *RNF144A*, *and VWA5A*) was observed in agreement with the Microarray and the RT-QPCR in the MEFs.

RT-qPCR assays were also conducted with human homologs to validate the genes that were believed to be regulated by *Mta1* in MEFs. This step of validation is performed with the *Mta1* knock down (*Mta1* siRNA) in MCF7compared with the non-target control siRNA. Similar differential gene expression levels were observed among all the genes (*VWA5A*, *EGR2*, *RNF144A*, *KLF15*, *and HMMR*) which are similar to the microarray expression profile (Figure 8).

## Discussion

This study represents a complete genome wide screen for possible target genes of a transcriptional co regulator, *Mta1*. In addition to identifying “bona fide” *Mta1* target genes, the influence of *p53* on *Mta1* gene regulation and molecular function has also been analyzed extensively. Emerging literature on *Mta1* clearly establishes bidirectional interplay between the oncogene, MTA1 and the tumor suppressor, P53. Although *Mta1* was found to be a component of the Nucleosome Remodeling and Deacetylase (NuRD) complex, recent studies from our laboratory establishes the functions of MTA1 in DNA-damage response in a P53-dependent and -independent manner [24], [30], [34]. This raises the compelling question of how *P53* influences the gene regulation and overall function of MTA1. We attempted to address this question using microarray approach. The first goal of identifying “bona fide” *Mta1* targets have been achieved by initial comparison of genes regulated by *Mta1* wild type and the *Mta1* knockout. Subsequently, this set of genes was compared with the set identified from the comparative study of *Mta1* knock out vs. *Mta1* re-expressed in the knock out MEFs. The common genes identified from both comparisons reflect the *Mta1* “bona fide” targets (Table 3 and Table S5). To achieve the goal of defining oncogenic gene profile of *Mta1*, we have mimicked most cancer scenarios i.e. loss of *P53* and the over expression of oncogenes such as *Mta1* and compared *P53* knock out and *P53* knockout in which there was over expression of *Mta1* gene. Interestingly, the identified 266 genes (Table 4) are mostly involved in DNA damage response. This is in agreement with the recently established *Mta1* function in DNA damage which is independent of *P53*. Together, these data further illustrate the possible genes that are regulated by *Mta1* “fail safe” mechanism which occurs due to loss of *P53*.

**Figure 9:**
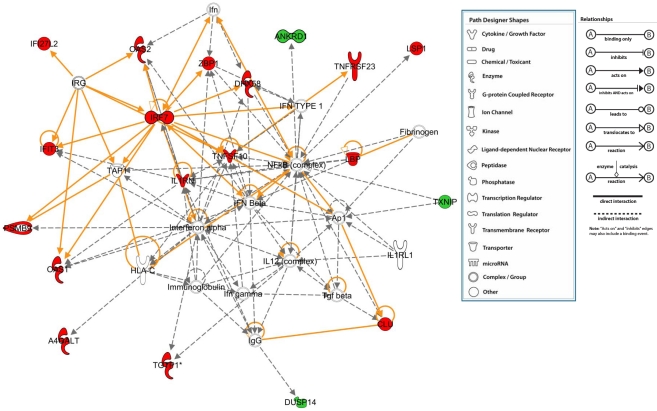
Gene network analysis shows that the genes regulated by *Mta1* in the presence of *P53* are involved in the networks associated with antimicrobial and inflammatory responses (statistically most significant network). When compared with the wild type MEFs, the genes that were up regulated in *Mta1* knockout MEFs are shown in red and the down regulated genes are shown in green. The network with the highest score from the IPA analysis was considered to be the most significant network and majority of the genes that were found in this network were up-regulated.

The gene ontology analysis again highlights the influence of *P53* on *Mta1* function. If *P53* is present, the targets regulated by *Mta1* play no remarkable role in the molecular functions such as catalytic activity or binding where as in the absence of *P53* about 39% of genes regulated by *Mta1* are involved in molecular functions, clearly indicating the influence of *P53* on *Mta1* gene regulation. Further extensive comparative analysis of all the data using IPA and networks reveals two distinct functional themes. In the presence of *P53*, the genes regulated by *Mta1* are mainly involved in the inflammatory response cancer and cellular movement. Whereas, in the absence of *P53* the genes regulated are predominantly related to cancer signaling reflecting the significance of *Mta1* in cancer. In agreement to the above mentioned observations, the top networks and pathways regulated by *Mta1* in the presence of *P53* appear to be antimicrobial response, inflammatory response and carbohydrate metabolism (Figure 9). For instance, most of the genes regulated by *Mta1* revolve around major complexes such as IRF7 (Interferon regulatory factor-7), which has been shown to play important role in the transcriptional activation of virus inducible cellular genes. In addition, as a part of innate antiviral immunity, the induction of systemic IFN takes place through IRF7 [42]. Another gene, Immunity-related GTPases (*IRG*) that play an important role in defense against intracellular pathogens and NFĸB complex which has been well ascertained to be regulated by *Mta1* were also found in the network. In agreement with our data recent studies suggest *MTA1* regulating its target genes either by acting as a corepressor [18], [19] or as transcriptional coactivator [20], [21] via interacting with RNA polymerase II. Together, these findings raise the possibility that *Mta1* may play a significant role in protecting regulation of innate immune response by directly modulating several pathways including NF-κB signaling [43]. Our data further supports this notion and emphasizes the role of *Mta1* in inflammation.

**Figure 10:**
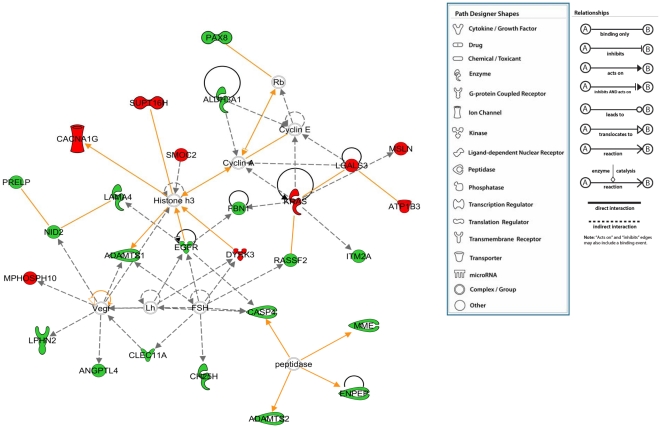
The genes differentially regulated (*p*-value<0.05 and fold change ≥±2.0) between the *P53* knock out and the *P53* knock out with *Mta1* over expressed MEFs (*Mta1* regulated genes independent of *P53*) were subjected to the network analysis and the most significant network found is associated with cancer and genetic disorders. Most of the genes in the network were found to be down regulated in *P53*-KO/*Mta1* when compared with *P53*-KO. All the upregulated genes are shown in red and the down regulated genes are shown in green color.

In contrast to that, in the absence of *P53*, *Mta1* target genes appear to be mainly involved in cancer and genetic disorders (Figure 10). For instance, many direct and indirect interactions were found with well studied cancer molecules such as Kirsten rat sarcoma viral oncogene homolog (KRAS) [44], [45], epidermal growth factor receptor (EGFR) [46]–[48] and vascular endothelial growth factor (VEGF) [49], [50] further highlighting the *P53*-independent central functions of *Mta1* in metastasis and cancer. Thus, MTA1 appears to be playing distinct molecular functions depending on the status of *P53*. In summary, our data presents complete gene profiling of *Mta1* in the presence and absence of *P53* representing a new resource and guide for future area of *Mta1* research which is unexplored but comprises several key elements that could be employed in the development of anticancer therapeutics and identification of novel functions of *Mta1*.

## Materials and Methods

### Cell Culture

Wild type Murine Embryonic Fibroblasts (MEFs) and *Mta1*-KO MEFs were obtained as described previously [35]. *P53* knock out MEFs were kindly provided by Dr. G. Lozano (M.D. Anderson Cancer Center, Houston, TX). MEFs and MCF7 (Michigan Cancer Foundation-7 human breast cancer cell line) were cultured in DMEM/F-12 medium containing 10% fetal bovine serum (FBS-Difco Laboratories, Detroit, Michigan) and 1% antibiotic-antimycotic solution in a humidified 5% CO_2_ at 37°C. Cell culture reagents were purchased from Invitrogen (Carlsbad, CA). *Mta1^+/+^*, *Mta1^−/−^*, *Mta1^−/−^/Mta1*, *p53^−/−^ and p53^−/−^/Mta1* MEFs have been described previously [23], [24]. Human MTA1 and non target control siRNA smart pools were obtained from Dharmacon, Inc. MCF7 cells were obtained from ATCC (ATCC number: HTB-22) Transfection into MCF7 cells was performed with Oligofectamine reagent (Invitrogen) and manufacturer's instructions were followed. Cells were collected after 36–48 hours after transfection.

### RNA Extraction & Microarray Gene Expression Arrays

Microarray gene expression assays have been performed as described previously [30]. In summary total RNA was extracted from the cells using Trizol (Invitrogen, Carlsbad, CA) and the manufacturer's protocol was followed. The quality and the concentrations of the extracted RNA were checked using the Nano-Drop (Thermo scientific). RNA was then purified using RNeasy Mini Kit (Qiagen, Valencia, CA) and the integrity was tested on 6000 NanoChips using Agilent 2100 Bioanalyzer (Agilent Technologies, Santa Clara, CA). Finally, Affymetrix Mouse Exon 1.0 ST arrays were used for the hybridization, arrays were scanned and the expression data was obtained in the form of .CEL files.

### Microarray data analysis

To analyze the data generated from the microarray experiments we used GeneSpring GX 10.0.2 (Agilent technologies, Inc) software package for the data Quality Control and the statistical analysis of the microarray data. The method of Benjamini and Hochberg was applied for the multiple corrections with a *p*-value cut-off of 0.05 and fold change ≥±2.0 to obtain the statistically significant genes. Heat maps for individual arrays were generated and the hierarchical cluster analysis was performed using MeV 4.5 [51]. Gene Ontology analysis was performed using GeneSpring GX 10.0.2 (Agilent Technologies). All data is MIAME compliant and the raw data has been deposited in Gene Expression Omnibus (GEO) as detailed on the Microarray Gene expression Data Society (MGED) society website (http://www.mged.org/Workgroups/MIAME/miame.html).

### Functional and Network Analysis

We used the Ingenuity pathway Analysis (Ingenuity Systems, Inc) to find the statistically significant pathways, functions and the networks in which the identified genes regulated by *Mta1*are possibly involved. Fischer's exact test was used to identify the significant functions and pathways represented within the respective gene sets.

### RT-qPCR

RT-qPCR analysis was performed following the protocol described previously [23], [24]. In order to validate the microarray data we selected candidate genes and RT-qPCR assays were conducted (all the primers used in this study are listed in Table S10). 2 µg of the total RNA was used for the first strand cDNA synthesis using the superscript III First-strand sys kit (Invitrogen), according to the manufacturer's instructions in 21-µl reactions. These reactions were diluted 1∶10 with nuclease-free water. Duplicates of the qPCR contained 2-µl of the first strand cDNA, 1-µl of the intron-spanning primers specific for that particular mRNA sample, 2 µl of nuclease free water and 5 µl of SYBR Green (Bio-Rad, Hercules, CA). Reactions (10 µl) were run in 96-well optical plates (Bio-Rad, Hercules, CA). Average threshold cycle (Ct) values of 18S mRNA (chosen as normalizer) were subtracted from the corresponding average Ct values of a target mRNA to obtain ΔCt values. The relative RNA levels were then expressed as 2^−Δct^.

## Supporting Information

Figure S1 Western blot assay of the five samples (WT, Mta1-KO, Mta1-KO/Mta1, P53-KO, and P53-KO/Mta1) was performed using Mta1 antibody as described previously [30] and the levels of Mta1 in all the five conditions are shown. α-Tubulin was used as the internal control.
(TIF)

Table S1 Complete list of the differentially expressed probe sets on the Affymetrix Mouse Exon 1.0 ST arrays between the wild type and the *Mta1* knock out MEFs.
(XLS)

Table S2 The statistical summary of the log 2 ratio values for the differentially expressed probe sets on the Affymetrix Mouse Exon 1.0 ST arrays between the wild type and the *Mta1* knock out MEFs.
(DOC)

Table S3 Complete list of the differentially expressed probe sets on the Affymetrix Mouse Exon 1.0 ST arrays between the *Mta1* knockout (*Mta1*-KO) MEFs and *Mta1* reintroduced into the knock out MEFs (*Mta1*-KO/*Mta1*).
(XLS)

Table S4 The statistical summary of the log 2 ratio values for the differentially expressed probe sets on the Affymetrix Mouse Exon 1.0 ST arrays between the *Mta1* knockout (*Mta1*-KO) MEFs and *Mta1* reintroduced into the knock out MEFs (*Mta1*-KO/*Mta1*).
(DOC)

Table S5 The ‘Bona fide’ genes that are regulated by *Mta1*.
(DOC)

Table S6 Complete list of the differentially expressed probe sets on the Affymetrix Mouse Exon 1.0 ST arrays between the *P53* knock out (*P53*-KO) and *P53* knockout MEFs with over expression of *Mta1*(*P53*-KO/*Mta1*).
(XLS)

Table S7 The statistical summary of the log 2 ratio values for the differentially expressed probe sets on the Affymetrix Mouse Exon 1.0 ST arrays in MEFs between the *P53* knockout (*P53*-KO) and *P53* knockout MEFs with over expression of *Mta1* (*P53*-KO/*Mta1*).
(DOC)

Table S8 Gene Ontology analysis of the genes differentially regulated between WT & *Mta1*-KO with ≥2.0 fold change.
(DOC)

Table S9 Gene Ontology analysis of the ‘bona fide’ *Mta1* regulated genes in the absence of *P53* with ≥2.0 fold change.
(DOC)

Table S10 Primer sequences for the candidate genes of *Mus Musculus* and *Homo sapiens* that were used in the RT-qPCR assays are shown.
(DOC)
