# Supplementary figures and images for: Gene Profiling of Mta1 Identifies Novel Gene Targets and Functions
Source: PLoS One. 2011 Feb 25;6(2):e17135. doi: 10.1371/journal.pone.0017135 (PMC3045407; doi:10.1371/journal.pone.0017135)

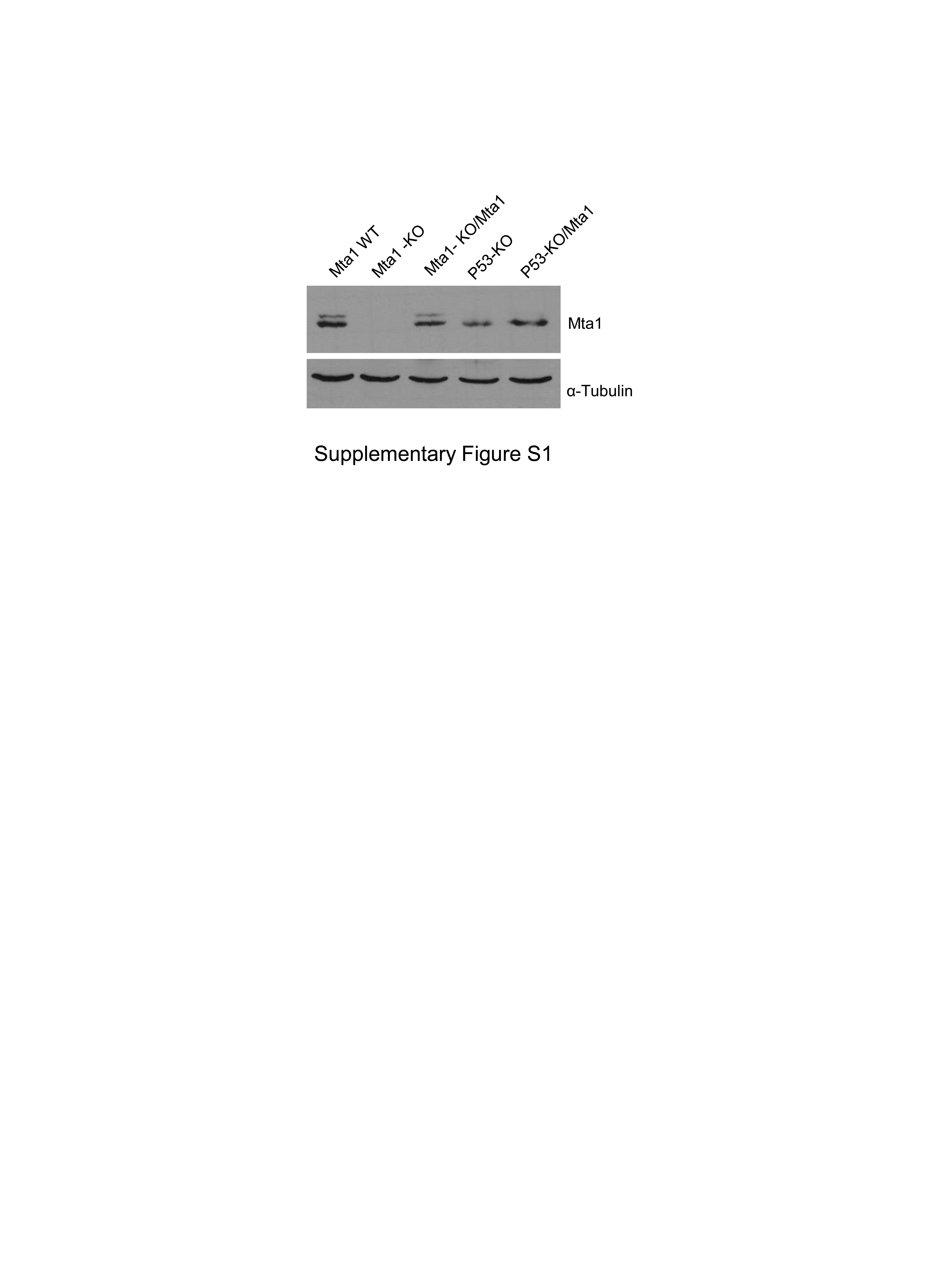

Supplement: Figure S1 — Western blot assay of the five samples (WT, Mta1-KO, Mta1-KO/Mta1, P53-KO, and P53-KO/Mta1) was performed using Mta1 antibody as described previously [30] and the levels of Mta1 in all the five conditions are shown. α-Tubulin was used as the internal control. (TIF) [file pone.0017135.s001.tif]
