# Supplementary material for: Gene Profiling of Mta1 Identifies Novel Gene Targets and Functions
Source: PLoS One. 2011 Feb 25;6(2):e17135. doi: 10.1371/journal.pone.0017135 (PMC3045407; doi:10.1371/journal.pone.0017135)
Supplement: Table S2 — The statistical summary of the log 2 ratio values for the differentially expressed probe sets on the Affymetrix Mouse Exon 1.0 ST arrays between the wild type and the Mta1 knock out MEFs. (DOC) [file pone.0017135.s003.doc]

**Table S2**: Statistical summary of the Significant differentially regulated genes between the MEFs Wild type and the *Mta1* knock out.

| Property | WT | *Mta1*-KO |
| --- | --- | --- |
| No. of Observations | 1124.00 | 1124.00 |
| No. of Missing Values | 0.00 | 0.00 |
| Minimum | -5.52 | -5.08 |
| Maximum | 4.55 | 5.09 |
| Mean | -0.26 | 0.42 |
| Trimmed Mean | -0.25 | 0.42 |
| Median | -0.23 | 0.34 |
| Std. Deviation | 1.19 | 1.22 |
| Trimmed Std. Deviation | 1.07 | 1.10 |
| No. Of Outliers | 28.00 | 47.00 |
| Percentile 1.0 | -3.74 | -2.99 |
| Percentile 5.0 | -2.19 | -1.38 |
| Percentile 10.0 | -1.57 | -1.03 |
| Percentile 25.0 | -0.95 | -0.26 |
| Percentile 50.0 | -0.23 | 0.34 |
| Percentile 75.0 | 0.57 | 1.10 |
| Percentile 90.0 | 1.17 | 1.92 |
| Percentile 95.0 | 1.48 | 2.56 |
| Percentile 99.0 | 2.68 | 3.79 |
