# Supplementary material for: Gene Profiling of Mta1 Identifies Novel Gene Targets and Functions
Source: PLoS One. 2011 Feb 25;6(2):e17135. doi: 10.1371/journal.pone.0017135 (PMC3045407; doi:10.1371/journal.pone.0017135)
Supplement: Table S4 — The statistical summary of the log 2 ratio values for the differentially expressed probe sets on the Affymetrix Mouse Exon 1.0 ST arrays between the Mta1 knockout (Mta1-KO) MEFs and Mta1 reintroduced into the knock out MEFs (Mta1-KO/Mta1). (DOC) [file pone.0017135.s005.doc]

**Table S4:** Statistical summary of the Significant differentially regulated genes between the *Mta1* knock out MEFs and the *Mta1* knock out MEFs with *Mta1*transfected back.

| **Property** | ***Mta1*-KO** | ***Mta1*-KO/*Mta1*** |
| --- | --- | --- |
| **No. of Observations** | 184.00 | 184.00 |
| **No. of Missing Values** | 0.00 | 0.00 |
| **Minimum** | -5.08 | -3.35 |
| **Maximum** | 4.65 | 3.90 |
| **Mean** | 0.84 | 0.28 |
| **Trimmed Mean** | 0.85 | 0.28 |
| **Median** | 1.04 | 0.08 |
| **Std. Deviation** | 1.45 | 0.99 |
| **Trimmed Std. Deviation** | 1.36 | 0.92 |
| **No. Of Outliers** | 5.00 | 11.00 |
| **Percentile 1.0** | -3.61 | -1.90 |
| **Percentile 5.0** | -1.44 | -1.05 |
| **Percentile 10.0** | -1.09 | -0.85 |
| **Percentile 25.0** | 0.00 | -0.24 |
| **Percentile 50.0** | 1.04 | 0.08 |
| **Percentile 75.0** | 1.61 | 0.73 |
| **Percentile 90.0** | 2.80 | 1.60 |
| **Percentile 95.0** | 3.22 | 2.25 |
| **Percentile 99.0** | 4.18 | 3.27 |
