# Supplementary material for: Gene Profiling of Mta1 Identifies Novel Gene Targets and Functions
Source: PLoS One. 2011 Feb 25;6(2):e17135. doi: 10.1371/journal.pone.0017135 (PMC3045407; doi:10.1371/journal.pone.0017135)
Supplement: Table S5 — The ‘Bona fide’ genes that are regulated by Mta1. (DOC) [file pone.0017135.s006.doc]

**Supplementary Table S5:** Bona fide *Mta1* regulated genes in the presence of *P53* identified from the Affymetrix Mouse Exon 1.0 ST Arrays.

| **Gene Symbol** | **WT vs. *Mta1*-KO** | | ***Mta1*-KO vs. *Mta1*-KO/*Mta1*** | |
| --- | --- | --- | --- | --- |
|  | **Fold Change** | **Regulation** | **Fold Change** | **Regulation** |
| Tgtp | 5.96 | up | 2.72 | down |
| Trib2 | 5.94 | up | 2.12 | down |
| Clu* | 5.67 | up | 3.1 | down |
| Cbr2* | 5.6 | up | 6.01 | down |
| Cx3cl1 | 5.52 | up | 2.22 | down |
| Pla2g7 | 5.47 | up | 2.24 | down |
| Ddah1 | 5.18 | down | 2.3 | up |
| Cyp2f2 | 5.1 | up | 3.65 | down |
| Phf17 | 4.92 | down | 2.48 | up |
| Glipr1 | 4.91 | up | 4.36 | down |
| Adam23 | 4.88 | up | 2.35 | down |
| Ctso | 4.82 | up | 2.69 | down |
| Iba2 | 4.64 | down | 2.41 | up |
| Aox1 | 4.55 | up | 2.15 | down |
| Txnip* | 4.51 | up | 2.79 | down |
| Aldh1a7 | 4.3 | up | 2.35 | up |
| Cxcl5* | 4.3 | down | 2.82 | down |
| Tnfaip6 | 4.2 | up | 2.32 | down |
| Wisp2* | 4.14 | up | 2.24 | down |
| Pcdhb4 | 4.12 | up | 2.15 | down |
| Scara5* | 3.97 | up | 2.91 | down |
| Loh11cr2a | 3.89 | up | 2.23 | down |
| Jag1 | 3.82 | up | 2.07 | down |
| Dna2 | 3.79 | down | 2.18 | up |
| Olfml3* | 3.74 | up | 2.36 | down |
| Sned1* | 3.68 | up | 2.25 | down |
| Tnfsf10 | 3.56 | up | 3.05 | down |
| Matn2 | 3.51 | up | 3 | down |
| Mmp19* | 3.5 | up | 2.38 | down |
| Dusp14 | 3.46 | down | 2.57 | up |
| Cdc14b | 3.46 | down | 2.75 | up |
| Loxl3 | 3.43 | up | 2.04 | down |
| Mylk | 3.41 | up | 2.02 | down |
| Npy1r* | 3.37 | down | 2.09 | up |
| Vnn1* | 3.26 | up | 2.36 | down |
| Nqo1 | 3.24 | up | 2.91 | down |
| Hist1h2bc | 3.18 | up | 3.91 | down |
| Leprel1 | 3.13 | up | 3.04 | down |
| Egr2 | 3.09 | up | 2.22 | down |
| Siae | 3.09 | up | 2.33 | down |
| Stard5 | 2.96 | up | 2.26 | down |
| Mmp9 | 2.96 | up | 2.65 | down |
| Pdlim1 | 2.93 | up | 2.06 | up |
| C1rl | 2.92 | up | 2.23 | down |
| Sgce | 2.91 | up | 3.04 | up |
| Cacna1g* | 2.9 | up | 2.25 | down |
| AU018778 | 2.9 | up | 2.56 | down |
| Gdpd1 | 2.83 | down | 2.36 | up |
| Lsp1 | 2.78 | up | 2.16 | down |
| Clec11a* | 2.75 | down | 2.71 | down |
| Ap1s2* | 2.74 | down | 2.04 | up |
| Ankrd1 | 2.68 | down | 2.73 | up |
| Hs6st2 | 2.62 | down | 2.48 | Up |
| **Gene Symbol** | **WT vs. *Mta1*-KO** | | ***Mta1*-KO vs. *Mta1*-KO/*Mta1*** | |
|  | **Fold Change** | **Regulation** | **Fold Change** | **Regulation** |
| Npr3* | 2.6 | down | 3.74 | up |
| Sesn3* | 2.53 | up | 2.15 | down |
| Sspn | 2.52 | up | 2.73 | down |
| Tmem45a | 2.52 | down | 2.18 | up |
| Inhba | 2.47 | down | 2.27 | up |
| Tnfrsf23* | 2.45 | up | 2.62 | up |
| D0H4S114 | 2.44 | up | 3.57 | down |
| Ces3 | 2.43 | up | 2.11 | down |
| Boc* | 2.42 | up | 2.76 | down |
| Fetub | 2.4 | up | 2.23 | down |
| Lrrc17 | 2.39 | up | 2.98 | down |
| Rspo2* | 2.38 | up | 2 | down |
| Usp53 | 2.35 | down | 2.12 | up |
| 4932415G12Rik | 2.34 | up | 2.17 | down |
| Fbln5 | 2.33 | up | 2.01 | down |
| Nedd9 | 2.33 | up | 2.02 | down |
| C76566 | 2.29 | up | 2.11 | down |
| Spon2* | 2.27 | up | 3.26 | down |
| Epha7 | 2.2 | down | 2.83 | up |
| Sepw1 | 2.19 | up | 2.02 | down |
| Bphl | 2.15 | up | 2.26 | down |
| 1700010C24Rik | 2.13 | up | 2.09 | down |
| Gstt1 | 2.13 | up | 3.64 | down |
| Vav3 | 2.09 | down | 2.75 | up |
| Crkrs | 2.04 | down | 2.06 | up |
