# Supplementary material for: Gene Profiling of Mta1 Identifies Novel Gene Targets and Functions
Source: PLoS One. 2011 Feb 25;6(2):e17135. doi: 10.1371/journal.pone.0017135 (PMC3045407; doi:10.1371/journal.pone.0017135)
Supplement: Table S7 — The statistical summary of the log 2 ratio values for the differentially expressed probe sets on the Affymetrix Mouse Exon 1.0 ST arrays in MEFs between the P53 knockout (P53-KO) and P53 knockout MEFs with over expression of Mta1 (P53-KO/Mta1). (DOC) [file pone.0017135.s008.doc]

**Table S6: Statistical summary (log2 values) of the Significant differentially regulated genes between the *P53* knock out MEFs and the *P53* knock out MEFs with *Mta1*transfected.**

| **Property** | ***P53*-KO** | ***P53*-KO/*Mta1*** |
| --- | --- | --- |
| **No. of Observations** | 266.00 | 266.00 |
| **No. of Missing Values** | 0.00 | 0.00 |
| **Minimum** | -2.17 | -3.99 |
| **Maximum** | 4.64 | 5.08 |
| **Mean** | 0.92 | -0.03 |
| **Trimmed Mean** | 0.92 | -0.03 |
| **Median** | 0.98 | -0.05 |
| **Std. Deviation** | 1.22 | 1.04 |
| **Trimmed Std. Deviation** | 1.15 | 0.92 |
| **No. Of Outliers** | 2.00 | 10.00 |
| **Percentile 1.0** | -1.73 | -3.22 |
| **Percentile 5.0** | -1.17 | -1.57 |
| **Percentile 10.0** | -0.73 | -1.19 |
| **Percentile 25.0** | 0.02 | -0.62 |
| **Percentile 50.0** | 0.98 | -0.05 |
| **Percentile 75.0** | 1.66 | 0.54 |
| **Percentile 90.0** | 2.54 | 1.23 |
| **Percentile 95.0** | 3.04 | 1.73 |
| **Percentile 99.0** | 4.21 | 2.64 |
