# Supplementary material for: Gene Profiling of Mta1 Identifies Novel Gene Targets and Functions
Source: PLoS One. 2011 Feb 25;6(2):e17135. doi: 10.1371/journal.pone.0017135 (PMC3045407; doi:10.1371/journal.pone.0017135)
Supplement: Table S8 — Gene Ontology analysis of the genes differentially regulated between WT & Mta1-KO with ≥2.0 fold change. (DOC) [file pone.0017135.s009.doc]

**Supplementary Table S8: GO Analysis of the genes differentially regulated between the WT and *Mta1*-KO with ≥ 2.0 fold change**

| **GO ACCESSION** | **GO Term** | **p-value** | **Corrected p-value** | **Count in Selection** | **% Count in Selection** | **Count in Total** | **% Count in Total** |
| --- | --- | --- | --- | --- | --- | --- | --- |
|  |  |  |  |  |  |  |  |
| GO:0048731 | system development | 6.82E-09 | 1.14E-04 | 28 | 4.39 | 1637 | 9.17 |
| GO:0009887 | organ morphogenesis | 8.36E-09 | 1.27E-04 | 28 | 4.39 | 522 | 2.92 |
| GO:0006952 | defense response | 9.08E-09 | 1.30E-04 | 33 | 5.17 | 421 | 2.36 |
| GO:0048856 | anatomical structure development | 1.71E-08 | 2.32E-04 | 28 | 4.39 | 1799 | 10.08 |
| GO:0016337 | cell-cell adhesion | 3.55E-08 | 4.57E-04 | 23 | 3.61 | 204 | 1.14 |
| GO:0050793 | regulation of developmental process | 6.32E-08 | 7.09E-04 | 1 | 0.16 | 758 | 4.25 |
| GO:0045087 | innate immune response | 6.21E-08 | 7.09E-04 | 18 | 2.82 | 84 | 0.47 |
| GO:0006950 | response to stress | 6.24E-08 | 7.09E-04 | 43 | 6.74 | 975 | 5.46 |
| GO:0007275 | multicellular organismal development | 2.04E-07 | 0.00218996 | 84 | 13.17 | 2155 | 12.07 |
| GO:0042127 | regulation of cell proliferation | 6.02E-07 | 0.00621014 | 7 | 1.10 | 371 | 2.08 |
| GO:0007389 | pattern specification process | 7.12E-07 | 0.007057759 | 10 | 1.57 | 241 | 1.35 |
| GO:0007156 | homophilic cell adhesion | 9.76E-07 | 0.009318451 | 19 | 2.98 | 90 | 0.50 |
| GO:0019838 | growth factor binding | 1.20E-06 | 0.01068921 | 13 | 2.04 | 52 | 0.29 |
| GO:0007167 | enzyme linked receptor protein signaling pathway | 1.36E-06 | 0.011452871 | 18 | 2.82 | 237 | 1.33 |
| GO:0031225 | anchored to membrane | 1.72E-06 | 0.013836062 | 23 | 3.61 | 129 | 0.72 |
| GO:0007169 | transmembrane receptor protein tyrosine kinase signaling pathway | 3.24E-06 | 0.02385981 | 18 | 2.82 | 153 | 0.86 |
| GO:0030246 | carbohydrate binding | 4.18E-06 | 0.029914644 | 1 | 0.16 | 271 | 1.52 |
| GO:0005201 | extracellular matrix structural constituent | 7.58E-06 | 0.052793097 | 9 | 1.41 | 60 | 0.34 |
| GO:0004908 | interleukin-1 receptor activity | 1.34E-05 | 0.088519424 | 5 | 0.78 | 7 | 0.04 |
| GO:0019966 | interleukin-1 binding | 1.34E-05 | 0.088519424 | 5 | 0.78 | 7 | 0.04 |
| GO:0007160 | cell-matrix adhesion | 1.37E-05 | 0.08854531 | 12 | 1.88 | 55 | 0.31 |
| GO:0005520 | insulin-like growth factor binding | 1.45E-05 | 0.090989135 | 8 | 1.25 | 21 | 0.12 |
