# Supplementary material for: Gene Profiling of Mta1 Identifies Novel Gene Targets and Functions
Source: PLoS One. 2011 Feb 25;6(2):e17135. doi: 10.1371/journal.pone.0017135 (PMC3045407; doi:10.1371/journal.pone.0017135)
Supplement: Table S9 — Gene Ontology analysis of the ‘bona fide’ Mta1 regulated genes in the absence of P53 with ≥2.0 fold change. (DOC) [file pone.0017135.s010.doc]

**Supplementary Table S9:** GO Analysis of the bona fide *Mta1* regulated genes in the absence of *P53* with ≥ 2.0 fold change

| **GO ACCESSION** | **GO Term** | **p-value** | **corrected p-value** | **Count in Selection** | **%Count in Selection** | **Count in Total** | **% Count in Total** |
| --- | --- | --- | --- | --- | --- | --- | --- |

| GO:0044266 | multicellular organismal macromolecule catabolic process | 1.99E-06 | 0.0264874 | 5 | 3.65 | 16 | 0.09 |
| --- | --- | --- | --- | --- | --- | --- | --- |
| GO:0044243 | multicellular organismal catabolic process | 1.99E-06 | 0.0264874 | 5 | 3.65 | 16 | 0.09 |
| GO:0044254 | multicellular organismal protein catabolic process | 1.99E-06 | 0.0264874 | 5 | 3.65 | 16 | 0.09 |
| GO:0044256 | protein digestion | 1.99E-06 | 0.0264874 | 5 | 3.65 | 16 | 0.09 |
| GO:0044236 | multicellular organismal metabolic process | 2.79E-06 | 0.03130538 | 5 | 3.65 | 17 | 0.10 |
| GO:0032963 | collagen metabolic process | 2.79E-06 | 0.03130538 | 5 | 3.65 | 17 | 0.10 |
| GO:0009611 | response to wounding | 3.68E-06 | 0.03952132 | 7 | 5.11 | 291 | 1.63 |
| GO:0019199 | transmembrane receptor protein kinase activity | 7.32E-06 | 0.07543227 | 7 | 5.11 | 72 | 0.40 |
