# Supplementary material for: Gene Profiling of Mta1 Identifies Novel Gene Targets and Functions
Source: PLoS One. 2011 Feb 25;6(2):e17135. doi: 10.1371/journal.pone.0017135 (PMC3045407; doi:10.1371/journal.pone.0017135)
Supplement: Table S10 — Primer sequences for the candidate genes of Mus Musculus and Homo sapiens that were used in the RT-qPCR assays are shown. (DOC) [file pone.0017135.s011.doc]

**Supplementary Table S7:** Primer Sequences used for the RT-qPCR Assays

***Mus Musculus***

| **Gene** | **Forward Sequence 5’-3’** | **Reverse Sequence 5’-3’** |
| --- | --- | --- |
| *Aw551984* | GGGCCCCTGGTGCATAGGGAT | TAGCGCTGTTGGGGTGTGCC |
| *Egr2* | GCCCAGCAAAACGCCAGTGC | AAGGGCTTGTGGCCCGTGTG |
| *Phf17* | TCGGTGGCCCGGACCCAT | CTGCTGCTGGGAAGGCGACC |
| *Hmmr* | AGCTCGCCCTGGCTGAGTTG | ATGGCTTGGGGGTGAGCAGC |
| *Klf15* | AGCACATGATGGCCCCGTGC | AAGCTGGGCGACCTTGACGC |
| *Rnf144a* | CGGCAACAGATCAGCTCCAGCC | AAGCAGCCCCGGGTACACCA |
| *Wnt5a* | TGCAACCCCTCACCCCCACA | AGTGTCCACCAGGGCTGCCA |
| *Egfr* | ATGCCGCCTCCTGGGCATCT | TGGCTGCCAAGTCACGGTGC |
| *Timp3* | TCCCGCCGGGCTACTTGGAA | GCGGGGACTGCCGCTCTTTT |
| *18S-*Mouse | CATGGCCGTTCTTAGTTGGT | GAACGCCACTTGTCCCTCTA |

***Homo sapiens***

| **Gene** | **Forward Sequence 5’-3’** | **Reverse Sequence 5’-3’** |
| --- | --- | --- |
| *EGR2* | CCCAGGCTCAGTCCAACCCCT | CCTCGCACAACCTGGAGACCC |
| *VWA5A* | GCCCAGGTCAGAGGTCTGCG | GCTCCCGGTGGAGGGTGAGT |
| *HMMR* | TTTGAAACCGGTAGGGAGTG | CCAGCTCCAGAAACTGAAGG |
| *KLF15* | GCTCGTCCCCTCCTCCGGTT | CCAGGACAGCCTGGCCCTCT |
| *RNF144A* | CGCGGGAGTCATTGGCTGGG | GCCACTGGACACAGGGCCAC |
| *18S-*HUMAN | CCGGAGCTAGGAATAATGGA | CCCTCTTAATCATGGCCTCA |
